# Supplementary material for: Dismantling the Component-Specific Effects of Yogic Breathing: Feasibility of a Fully Remote Three-Arm RCT with Virtual Laboratory Visits and Wearable Physiology
Source: Int J Environ Res Public Health. 2023 Feb 11;20(4):3180. doi: 10.3390/ijerph20043180 (PMC9958552; doi:10.3390/ijerph20043180)
Supplement: Supplementary file 1 [file ijerph-20-03180-s001.zip › ijerph-2102459-supplementary.pdf]

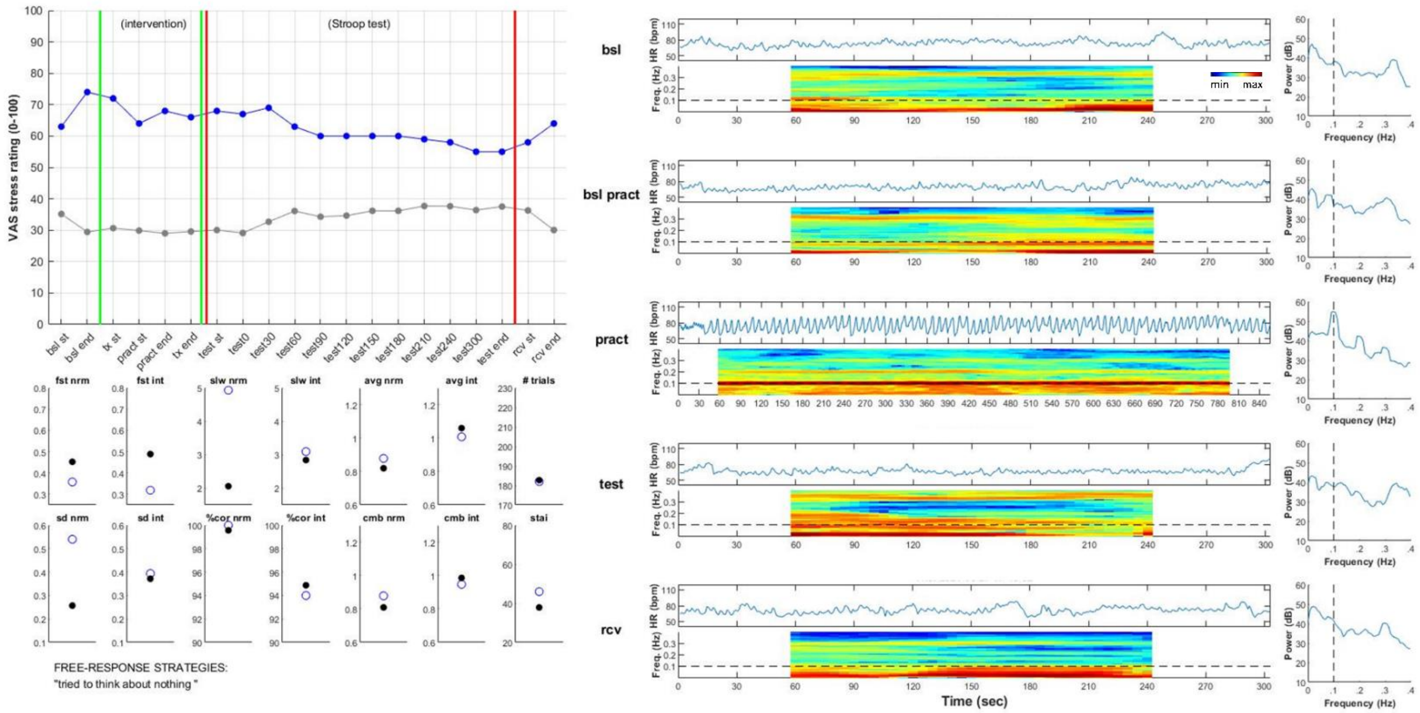

Supplementary Figure S1. Representative example of individual participant data from the virtual laboratory visit (VLV), including visual analog scale (VAS) ratings of momentary stress level across the visit, stress test performance, and heart rate recordings. In top left panel, grey line indicates average of all participants and blue line indicates individual participant. Stroop performance metrics on the bottom left include fast reaction time with color-word congruent (fst nrm, in seconds) and incongruent (fst int) trials, slowest reaction times (slw), average reaction times (avg), number of completed trials (# trials), standard deviation of the congruent (sd nrm) and incongruent (sd int) trials, percent correct for congruent (%cor nrm) and incongruent (%cor int) trials, combined average for congruent (cmb nrm) and incongruent (cmb int) trials, and State-Trait Anxiety Inventory score (stai) completed immediately following the Stroop test. Recording blocks on the right include baseline (bsl), baseline immediately preceding guided intervention practice (bsl pract), the guided practice (pract), Stroop test (test), and post-Stroop recovery (rcv).

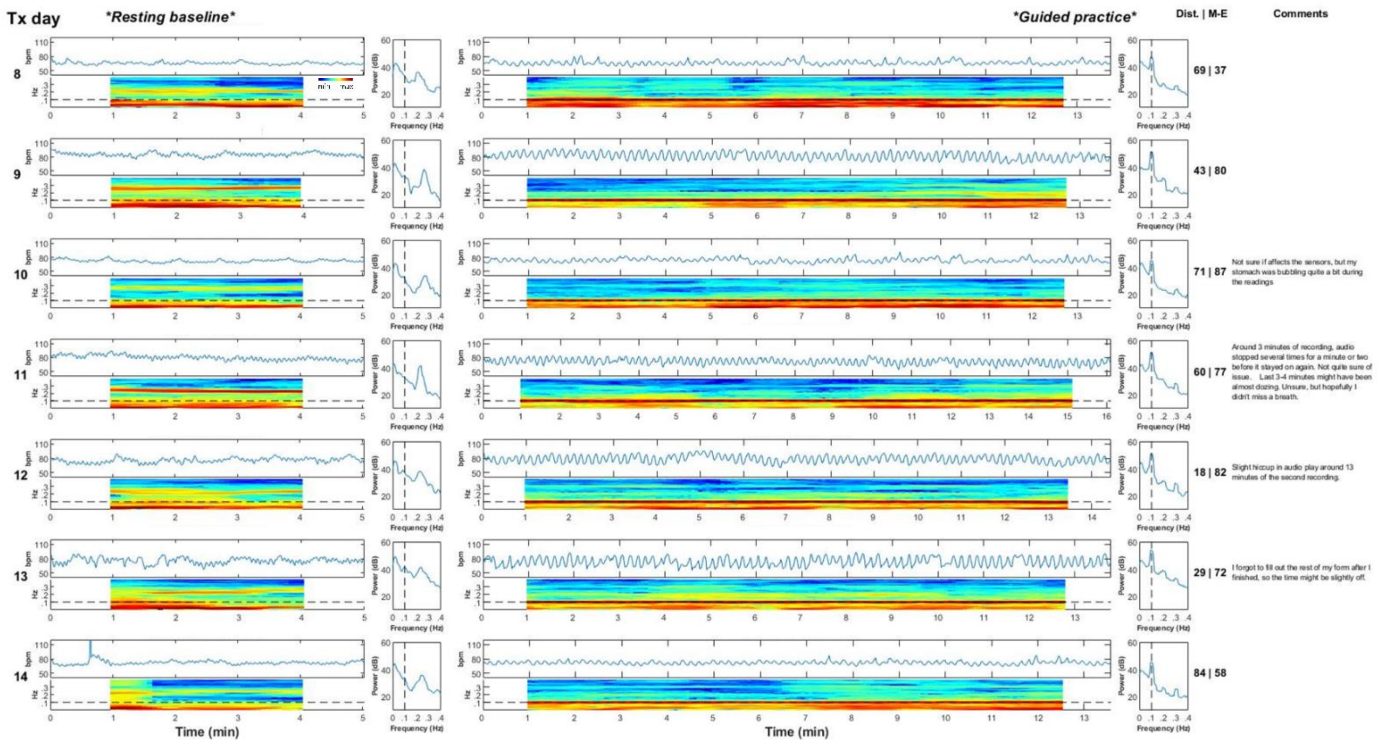

Supplementary Figure S2. Representative example of individual participant data for one week of daily practice logs and HR recordings. For each day (clustered row), HR timeseries is shown in the top panel and corresponding spectrogram in the bottom panel, with averaged spectral power shown to the right. Dashed horizontal and vertical lines indicate 0.1 Hz (6 bpm). Tx day, treatment/intervention day; Dist: 0-100 rating of distractions during practice; M-E: 0-100 rating of motivation-engagement.

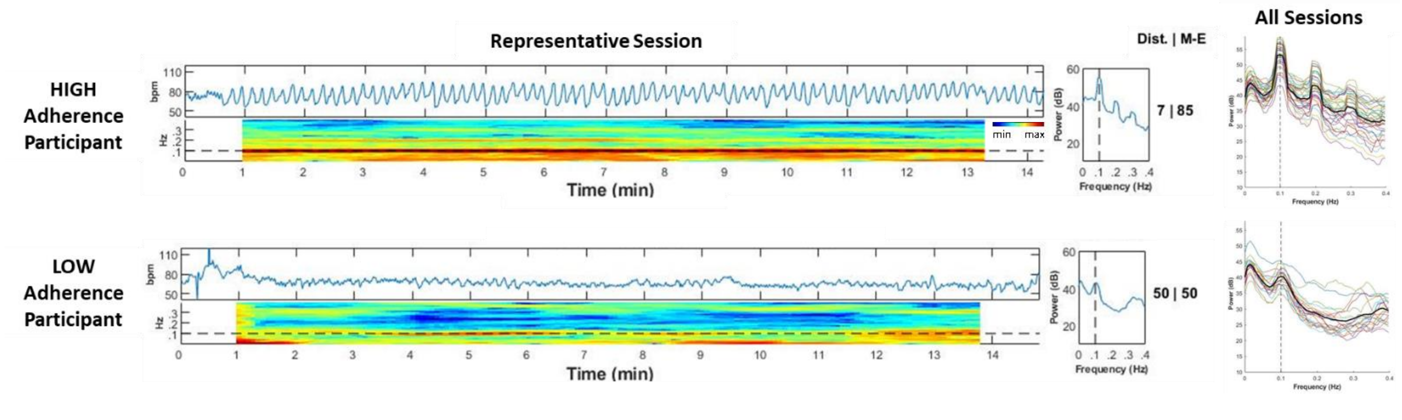

Supplementary Figure S3. Contrasting examples of a SPB participant who was highly adherent to the guided breathing rhythm throughout the study versus a SPB participant who was minimally adherent, as reflected by both self-report data and heart rate recordings. Dist: 0-100 rating of distractions during practice; M-E: 0-100 rating of motivation-engagement. Note that the low engagement participant frequently indicated a rating of 50, the default rating of the slider scale.

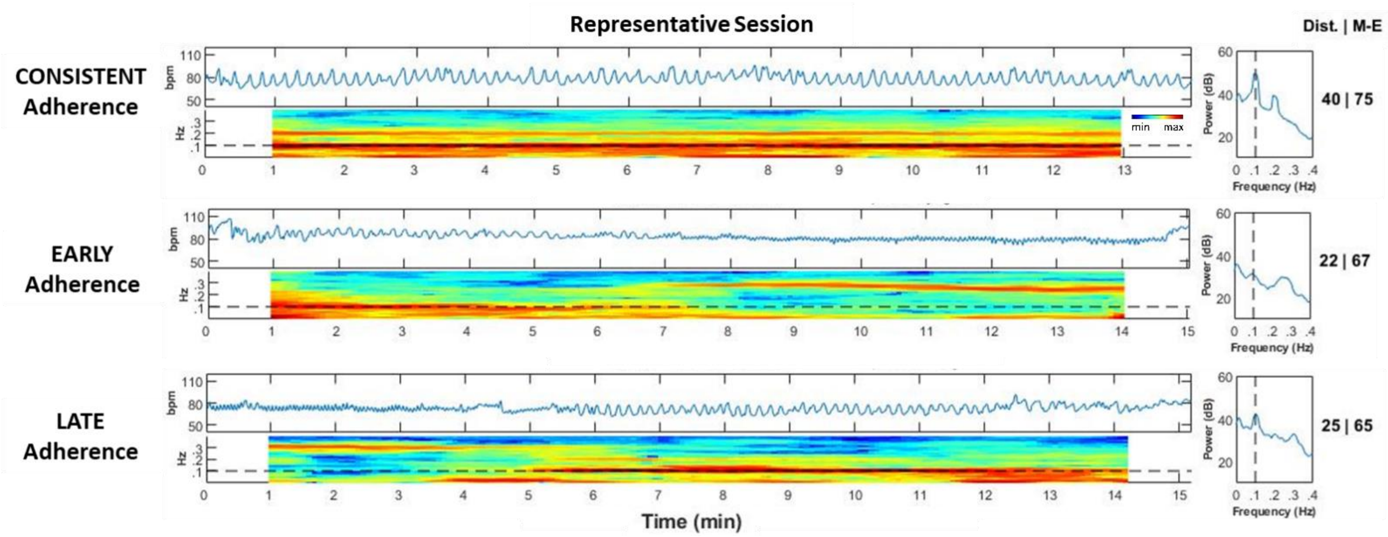

Supplementary Figure S4. Examples of varying timecourse patterns of adherence from an individual SPB participant. This participant was consistently adhered to the paced breathing pattern (6 bpm) for the entirety of the initial training session at VLV-Pre (top row), then demonstrated only partial adherence during other sessions, limited to the first half of session on training day 33 (middle) and second half on day 3 (bottom). Dist: 0-100 rating of distractions during practice; M-E: 0-100 rating of motivation-engagement.

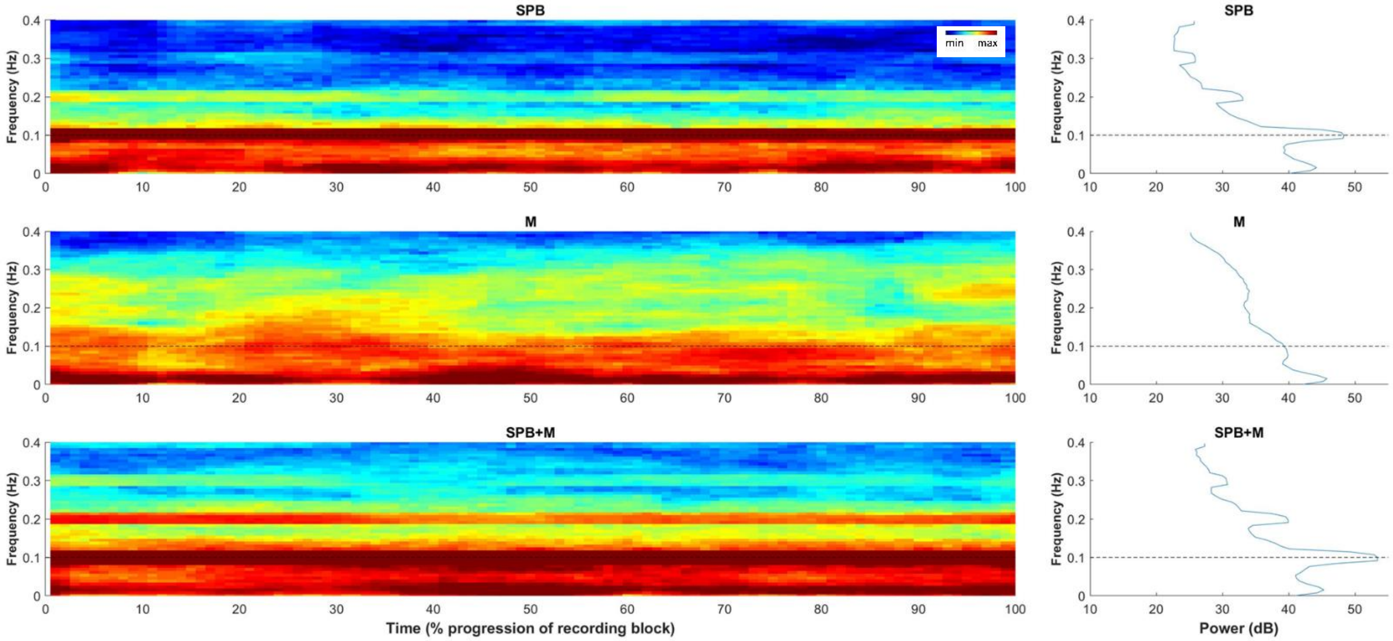

Supplementary Figure S5. Timecourse of HRV spectral power during practice sessions, plotted as the average across all participants and sessions for each group, normalized in the time domain to account for slightly varying recording times across sessions (despite equivalent 15-min durations of guided audio, a participant may have started and/or stopped the heart rate recording at varying times).

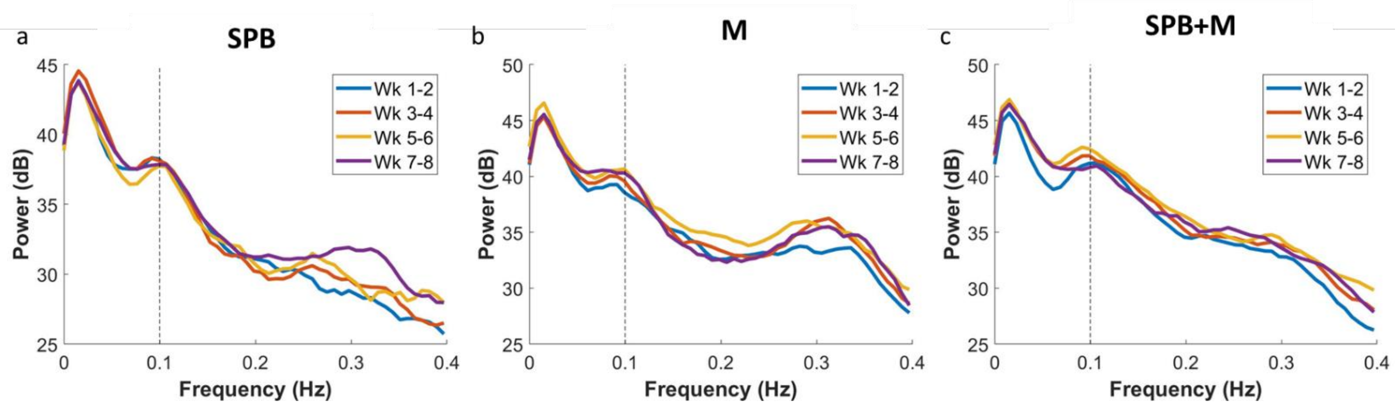

Supplementary Figure S6. HRV spectral power of daily resting baseline recordings from the 8-week training period, averaged across all available recordings in 2-week intervals for each group. Although there appeared to be an integration of the 6-bpm breathing rhythm during resting baseline at VLV-Post for SPB (Figure S3), there does not appear to be a progressive shift across the 8-week training period. Furthermore, all groups evidenced some amount of breathing near 6 bpm (0.1 Hz) in all of the 2-week intervals.
